# Caveats on COVID-19 herd immunity threshold: the Spain case

**DOI:** 10.1038/s41598-021-04440-z

**Published:** 2022-01-12

**Authors:** David García-García, Enrique Morales, Eva S. Fonfría, Isabel Vigo, Cesar Bordehore

**Affiliations:** 1 Department of Applied Mathematics, University of Alicante, Alicante, Spain; 2 Multidisciplinary Institute for Environmental Studies “Ramon Margalef”, University of Alicante, Alicante, Spain; 3 Department of Ecology, University of Alicante, Alicante, Spain

**Keywords:** Ecological epidemiology, Policy and public health in microbiology, Differential equations, Dynamical systems, Nonlinear dynamics, Population dynamics, Viral infection

## Abstract

After a year of living with the COVID-19 pandemic and its associated consequences, hope looms on the horizon thanks to vaccines. The question is what percentage of the population needs to be immune to reach herd immunity, that is to avoid future outbreaks. The answer depends on the basic reproductive number, *R*_0_, a key epidemiological parameter measuring the transmission capacity of a disease. In addition to the virus itself, *R*_0_ also depends on the characteristics of the population and their environment. Additionally, the estimate of *R*_0_ depends on the methodology used, the accuracy of data and the generation time distribution. This study aims to reflect on the difficulties surrounding *R*_0_ estimation, and provides Spain with a threshold for herd immunity, for which we considered the different combinations of all the factors that affect the *R*_0_ of the Spanish population. Estimates of *R*_0_ range from 1.39 to 3.10 for the ancestral SARS-CoV-2 variant, with the largest differences produced by the method chosen to estimate *R*_0_. With these values, the herd immunity threshold (HIT) ranges from 28.1 to 67.7%, which would have made 70% a realistic upper bound for Spain. However, the imposition of the delta variant (B.1.617.2 lineage) in late summer 2021 may have expanded the range of *R*_0_ to 4.02–8.96 and pushed the upper bound of the HIT to 90%.

## Introduction

On 11 March 2020, the World Health Organization declared the COVID-19 pandemic, and by 11 March 2021, 2.63 million people had died because of it^1^. However, although these are the published figures, there were probably many more undocumented virus related deaths that were not recorded due to lack of tests^2–4^. After a year of struggling, restrictions to lessen the spread of the virus, a downturn in the economy and the cost of human lives, most people are wondering when the pandemic will end. The year 2020 ended with the hopeful approval of some vaccines^5^, but how many people must be vaccinated to return to pre-pandemic life? The answer is quite complicated since vaccines do not provide 100% protection against infections^6,7^ nor fully block the transmissibility of the virus^8–10^. However, it is theoretically interesting to study when the *herd immunity threshold* (HIT) will be reached, if possible, under the assumptions that immune population (recovered and vaccinated people) get permanent immunisation against the different mutations of the SARS-CoV-2 virus and will not transmit the virus any further. In Spain, there is a general opinion that the HIT will be reached when 70% of the population becomes immune, which is not equivalent to 70% of vaccinated population in real life. Note that there is no single definition of HIT^11^ and this can lead to misunderstandings. In this study, HIT will refer to the minimum proportion of the immune population that will produce a monotonic decrease of new infections, even if restrictions are lifted and society returns to a pre-pandemic level of social contact. The question is how realistic is a HIT of 70% for Spain.

The HIT is usually defined in terms of the *effective reproduction number*, *R*_*e*_(*t*), which is the average number of secondary infections produced by an infected individual at time *t*. Any outbreak starts with *R*_*e*_ > 1, stabilizes with *R*_*e*_ = 1, and declines with *R*_*e*_ < 1. Therefore, the HIT will be reached when *R*_*e*_ = 1 and *R*_*e*_ < 1 afterwards. Given the number of susceptible individuals, that is, those that can get infected, *R*_*e*_(*t*) can be estimated in an unmitigated epidemic as^12,13^

1 $$ R_{e} \left( t \right) = R_{0} \cdot \frac{S\left( t \right)}{N}, $$

where *S*(*t*) is the number of susceptible individuals at time *t*; *N* is the total number of the population; and *R*_0_ is the *basic reproductive number*, that is, the expected number of secondary infections produced by an infected individual in a population where all individuals are susceptible and there are no measures to reduce transmission^12,14^. The proportion of susceptible, *S*(*t*)/*N*, can be written as 1 − *q*, where *q* is the proportion of immune population. Then, if *R*_*e*_(*t*) = 1 (and *R*_*e*_(*t*) < 1 afterwards), HIT equals *q* by definition. Replacing these equalities in Eq. (1) and operating, we get^15,16^

2 $$ HIT = 1 - \frac{1}{{R_{0} }}. $$

Note the direct relationship: the larger the *R*_0_, the larger the HIT; and that Eq. (2) makes sense only when *R*_0_ > 1, since for values *R*_0_ < 1 the disease will disappear naturally and the concept of HIT loses its sense. In Eq. (2) it is intrinsically assumed that recovered individuals cannot become susceptible again, that is, they cannot get re-infected nor transmit the virus after recovery. *R*_0_ is used to quantify the transmissibility of the virus, which depends on the virus itself and the characteristics of the population that is being infected. Regarding other infectious diseases, typical values of *R*_0_ are 0.9–2.1 for seasonal flu and 1.4–2.8 for the 1918 flu^17^, ~ 3 for SARS-CoV-1^18^ and < 0.8 for MERS^19^. For COVID-19 in 2020, a systematic review of 21 studies, mainly in China, found *R*_0_ ranging from 1.9 to 6.5^20^, which leads to HIT values between 47 and 84%. However, in 62% of these studies, the *R*_0_ was between 2 and 3 (HIT between 50 and 67%). In Western Europe in 2020, an average *R*_0_ was estimated at 2.2 (95% CI = [1.9, 2.6])^21^, with a HIT value of 55% (95% CI = [47, 62]). Therefore, 70% is an upper bound of HIT in 2020 in most of the cited cases, but not in all.

Theoretically, *R*_0_ can only be observed at the very beginning of the pandemic, while the whole population are susceptible and no control measures are in force (e.g., social distancing, the use of masks, etc.). This is the case in the above-mentioned studies^20,21^. However, during the COVID-19 pandemic the virus has mutated into more transmissible variants, with a higher *R*_0_. In consequence, the HIT has been increasing during the course of the pandemic, but its estimated value cannot be directly updated because the new variants did not exist at the beginning of the pandemic when the *R*_0_ should have been observed.

This study encompasses a detailed analysis of the HIT of the ancestral variant, that was the dominant variant at the beginning of the pandemic, from different approaches and quantifies the influence of three key factors: (1) source/quality of data; (2) infectiousness evolution over time; and (3) methodology to estimate *R*_0_. Finally, we indirectly estimate the *R*_0_ of the current dominant variants using Eq. (1) and comparisons between *R*_*e*_ values of several variants. The HIT values derived from these new *R*_0_ estimates are discussed in the last section.

## Data

Three COVID-19 daily infection datasets for Spain were used, from 1 January to 29 November 2020: (1) official infections published by the Instituto de Salud Carlos III (ISCIII,^22^); and Infections estimated with the REMEDID algorithm^23^ from (2) official COVID-19 deaths^22^, and (3) excess of all-causes deaths (ED) from European Mortality Monitoring surveillance system (MoMo,^24^). The REMEDID-derived infection data are more realistic than official infection data since they assimilate seroprevalence studies data^25^ and known dynamics of COVID-19 (see^23^, for further discussion). As the last national longitudinal seroprevalence study in Spain finished on 29 November 2021, our REMEDID time series has been estimated up to that date. This is not a limitation for this study since only data up to March 2020 will be used (see next section).

## Intrinsic growth rate

At the beginning of an outbreak the infections, *I*(*t*), increase exponentially^12,16^ and can be fitted to the model

3 $$ I\left( t \right) = ae^{rt} + \varepsilon \left( t \right), $$

where $\varepsilon \left( t \right)$ accounts for errors in the fitting; *t* is time; *a* is a positive number determining the point where the function crosses the ordinate axis, and then depends on where the origin of time has been set; and *r* is a positive number called *intrinsic growth rate* or *Malthusian number*, that defines the increasing rate of the exponential growth. *r* is usually the first property that epidemiologists estimate in an outbreak. The higher the *r*, the higher the speed in the increase of cases. When comparing diseases, *r* is an indicator of contagiousness, as is *R*_0_. In fact, with enough information about the latent and infectious periods, *r* (*t*^−1^ units) can be used to estimate *R*_0_ (dimensionless), although the relationship is not simple^26^. In the *latent period* (*exposed* in a Susceptible-Exposed-Infected-Recovered (SEIR) model), an infected individual cannot produce a secondary infection, unlike in the *infectious period*, where secondary infections may be produced.

**Figure 1:**
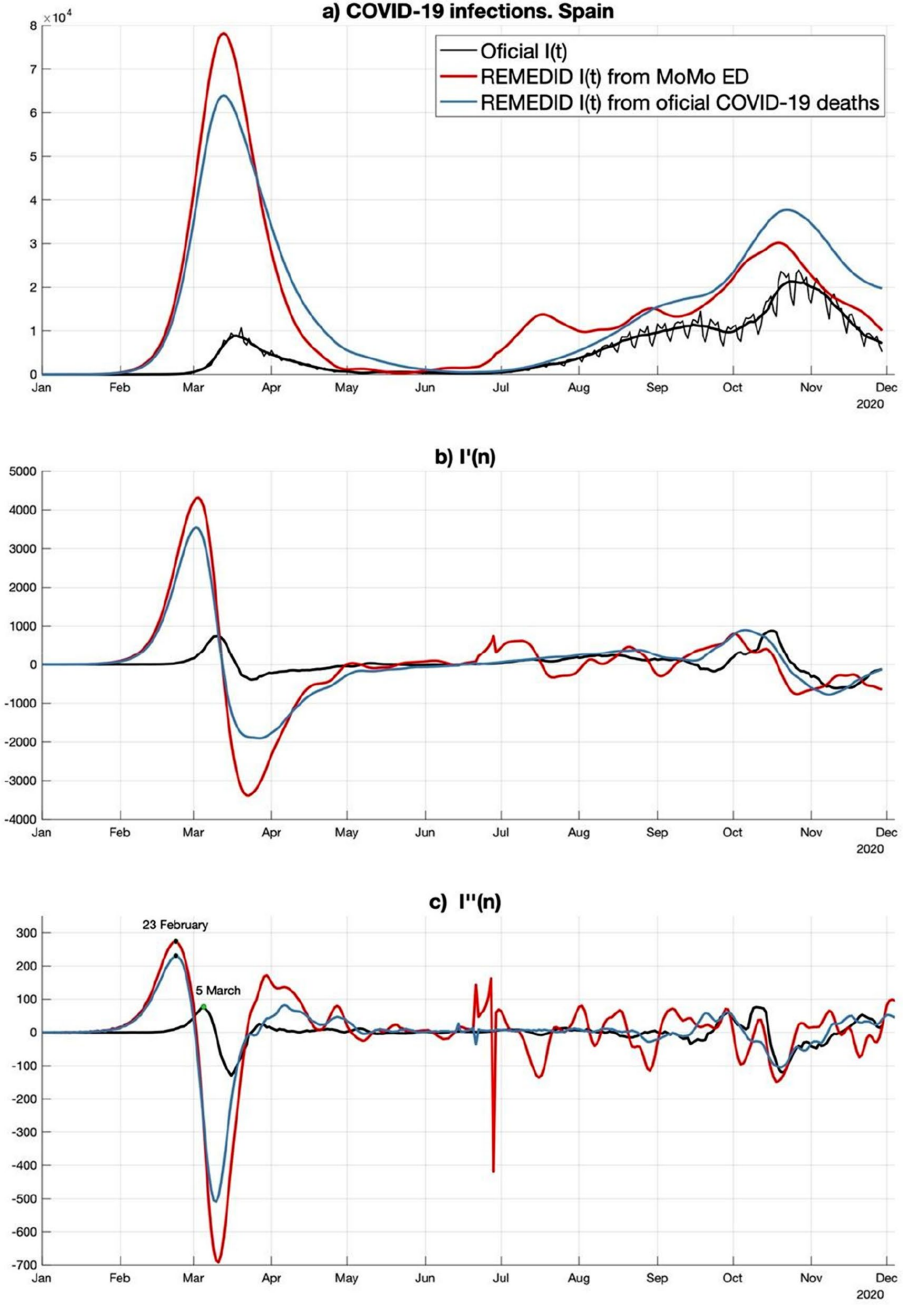
(**a**) Daily new infections: black thin line reflects official data, and thick black line is its 7-days moving average; red and blue lines are infections inferred from REMEDID methodology applied to MoMo excess of dead and to official COVID-19 deaths, respectively. (**b**) and (**c**) are the first and second discrete derivative of time series shown in (**a**). Official *I*′(*n*) (*I*″(*n*)) is estimated from the 7-days running mean of official *I*(*n*) (*I*′(*n*)). Panels (**b**) and (**c**) show the smoothed versions of *I*′(*n*) and *I*″(*n*), respectively.

**Figure 2:**
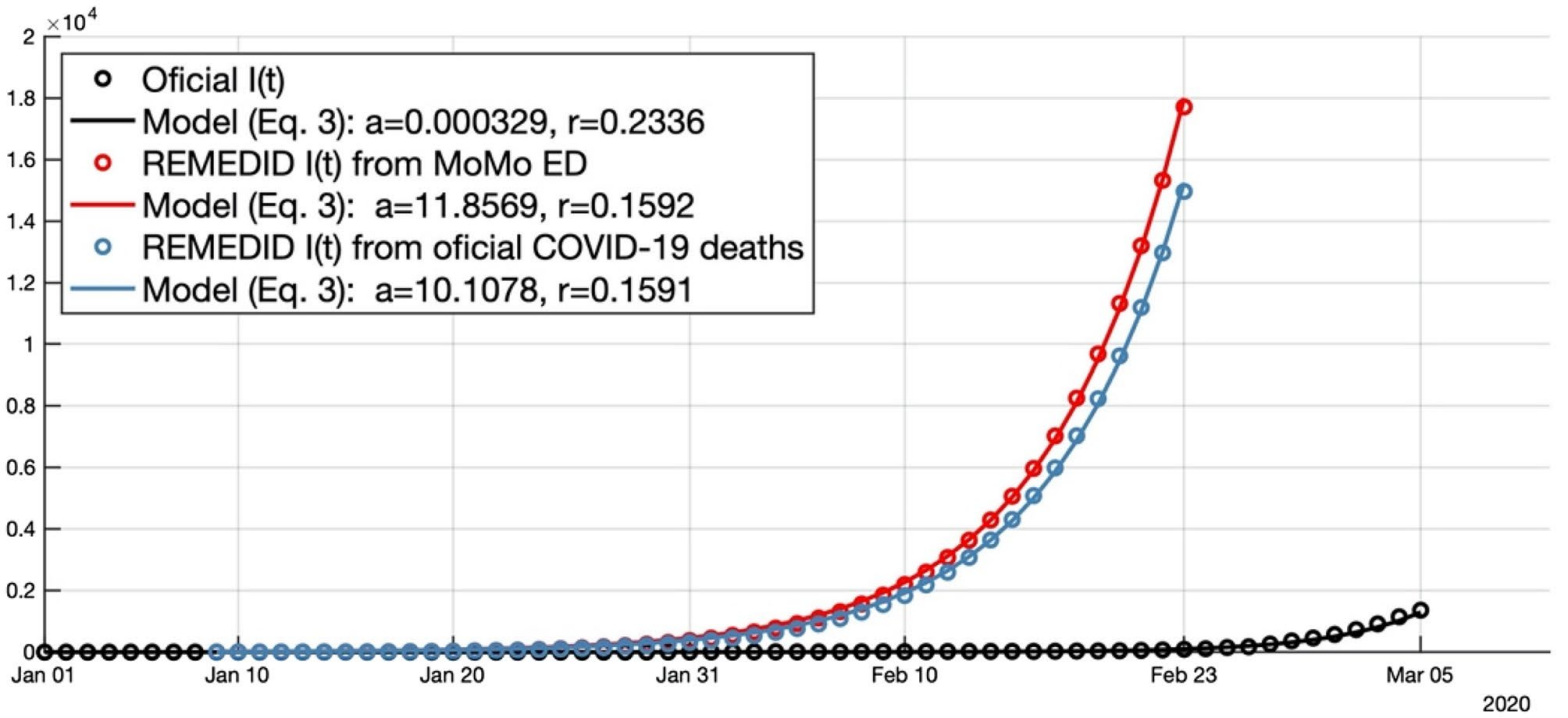
Daily new infections from official data (black dots) for a period of time of 65 days (1 January to 5 March 2020) and inferred from REMEDID applied to MoMo excess of dead (red dots) and official COVID-19 deaths (blue dots) for a period of time of 46 days (January 9 to February 23). Solid lines are the exponential fitting (Eq. 1) to them.

When estimating *r*, it must be kept in mind that *I*(*n*) (Fig. 1a), where *n* denotes time discretized in days, increases exponentially during a short period of time. Consequently, the first problem is to figure out the latest day, *n*_0_, before *I*(*n*) will abandon the strictly exponential growth because of the diminishing of the number of susceptible individuals. To estimate *n*_0_, we use the property that during the exponential growth *I*(*n*) is not only rising, but is accelerating with an increasing acceleration. Then, *n*_0_ is the day where the first maximum of *I*″(*n*), the second (discrete) derivative of *I*(*n*), is reached. For REMEDID *I*(*n*), from both official and MoMo data, *n*_0_ is 23 February 2020 (Fig. 1c). Figure 2 shows the least-squares best fit of Eq. (3) to REMEDID *I*(*n*) truncated at n_0_, whose parameters are:

1. *a* = 11.86 (95% CI = [11.01, 12.70]) and *r* = 0.1592 (95% CI = [0.1576, 0.1609]), when MoMo ED are used;
2. *a* = 10.11 (95% CI = [9.25, 10.96]) and *r* = 0.1591 (95% CI = [0.1571, 0.1610]), when official deaths are used.

Considering the Bonferroni correction, the difference between the two estimates of *r* has a CI = [− 0.0034, 0.0038], which has at least a 90% of confidence level. Since the CI includes the value 0, there is no evidence that these two parameters are different. Besides, a linearization of the model allows to perform a contrast of hypothesis on *r*, that confirms that there is no significant discrepancy between the two estimates of *r*. Then, REMEDID *I*(*n*) will be estimated from MoMo ED hereafter. Applying the same hypothesis for contrast, it can be observed that the *a* parameters are significatively different. However, since *a* value is not relevant to determine the growth rate, which is our aim here, we will not discuss its estimated values. If the same analysis were carried out with official *I*(*n*), which were not reliable at the beginning of the pandemic, we would get *r* = 0.2322 (95% CI = [0.2266, 0.2377]) and the end of the exponential growth on 5 March 2020. This value is significantly different, at least at 90% confidence level after Bonferroni correction, from the *r* estimated from any REMEDID *I*(*n*) since the CI of their differences do not include the 0. A contrast of hypothesis confirms this discrepancy. Note that despite the larger value of *r* from official *I*(*n*) the fitted exponential is smaller than those estimated from REMEDID *I*(*n*) (Fig. 2) because of the horizontal shift due to differences in the *a* parameter. The end of the exponential growth has been estimated from 7-days running averaged versions of *I*(*n*), *I′*(*n*), and *I*″(*n*) (Fig. 1a–c respectively). It has to be said that at the beginning of the outbreak, the official data underestimated the number of infections due to the low sampling capability.

## Estimates of ***R***_0_

### Generation time

**Figure 3:**
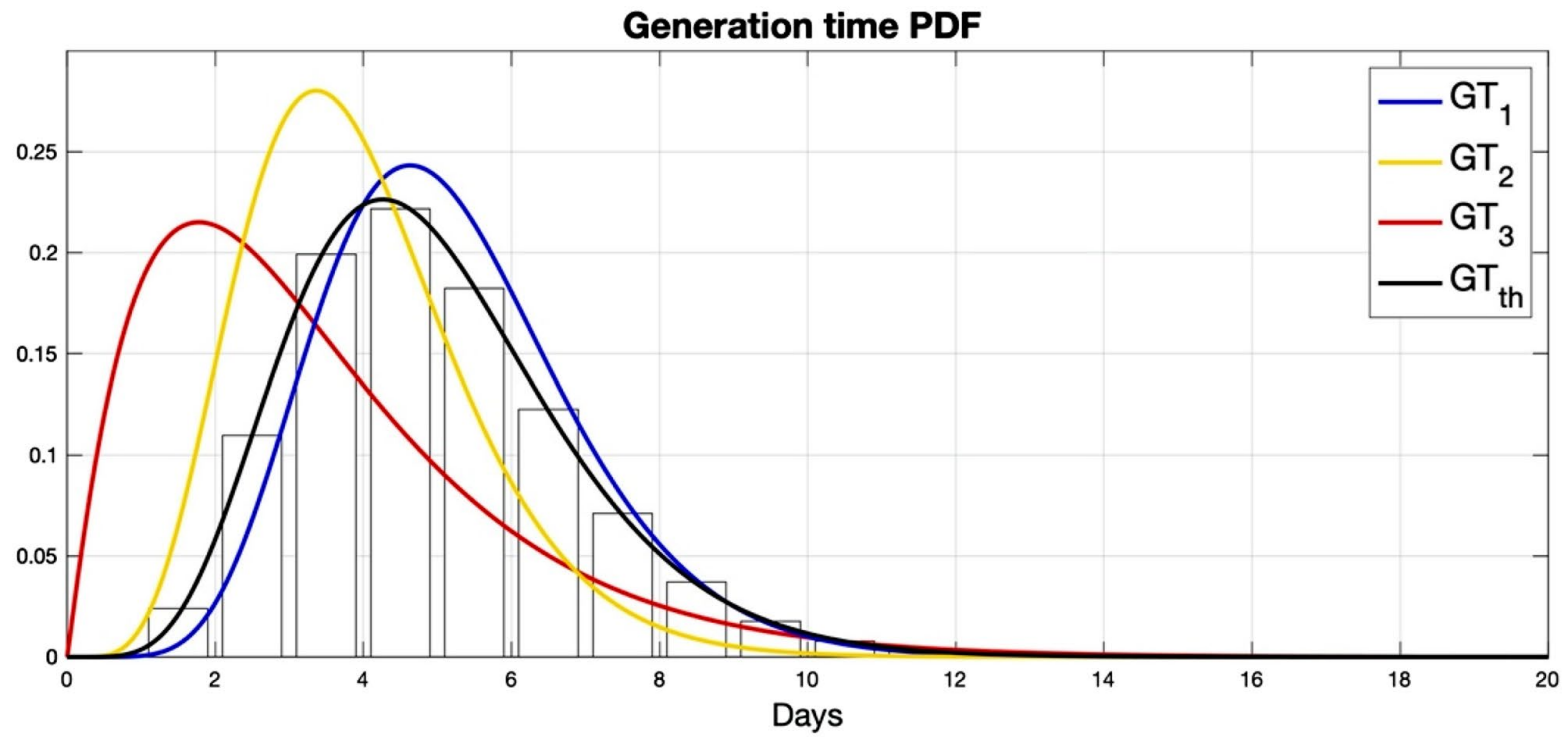
Probability density function of the generation time distribution, *f*_*GT*_(*t*), of *GT*_1_ (blue line; Singapore^27^), *GT*_2_ (yellow line; Tianjin^27^), *GT*_3_ (red line; Singapore^28^), and *GT*_*th*_ (black line; theoretical distribution). Bars are the discretized version, $\widetilde{{f_{GT} }}\left( n \right)$, of the PDF of *GT*_*th*_.

During the infectious period, an infected individual may produce a secondary infection. However, the individual’s infectiousness is not constant during the infectious period, but it can be approximated by the probability distribution of the *generation time* (GT), which accounts for the time between the infection of a primary case and the infection of a secondary case. Unfortunately, such distribution is not as easy to estimate as that of the *serial interval*, which accounts for the time between the onset of symptoms in a primary case to the onset of symptoms of a secondary case. This is because the time of infection is more difficult to detect than the time of symptoms onset. Ganyani et al.^27^ developed a methodology to estimate the distribution of the GT from the distributions of the incubation period and the serial interval. Assuming an incubation period following a gamma distribution with a mean of 5.2 days and a standard deviation (SD) of 2.8 days, they estimated the serial interval from 91 and 135 pairs of documented infector-infectee in Singapore and Tianjin (China). Then, they found that the GT followed a gamma distribution with mean = 5.20 (95% CI = [3.78, 6.78]) days and SD = 1.72 (95% CI = [0.91, 3.93]) for Singapore (hereafter *GT*_1_), and with mean = 3.95 (95% CI = [3.01, 4.91]) days and SD = 1.51 (95% CI = [0.74, 2.97]) for Tianjin (hereafter *GT*_2_). Ng et al.^28^ applied the same methodology to 209 pairs of infector-infectee in Singapore and determined a gamma distribution with mean = 3.44 (95% CI = [2.79, 4.11]) days and SD 2.39 (95% CI = [1.27, 3.45]; hereafter *GT*_3_). Figure 3 shows the probability density functions (PDF) of such distributions, *f*_*GT*_. The differences between them are remarkable. For example, the 54.5%, 81.0%, and 80.7% of the contagions are produced in a pre-symptomatic stage (in the first 5.2 days after primary infection) assuming *GT*_1_, *GT*_2_, and *GT*_3_, respectively.

Theoretically, assuming that the incubation periods of two individuals are independent and identically distributed, which is quite plausible, the expected/mean values of the GT and the serial interval should be equal^29,30^. The mean of the serial interval is easier to estimate than that of the GT. For that reason, we assume a mean serial interval as estimated from a meta-analysis of 13 studies involving a total of 964 pairs of infector-infectee, which is 4.99 days (95% CI = [4.17, 5.82])^31^, is more reliable than the aforementioned means of the GT. This value is within the error estimates of the means of *GT*_1_ and *GT*_2_, but not for *GT*_3_. Then, we construct a theoretical distribution for the GT that follows a gamma distribution (hereafter *GT*_*th*_) with mean = 4.99 days and SD = 1.88 days. This theoretical distribution can be seen in Fig. 3 and approximates the average PDF of three gamma distributions with mean = 4.99 and the SD of *GT*_1_, *GT*_2_, and *GT*_3_. We assume a conservative CI = [1.51, 2.39] for the theoretical SD, defined with the minimum and maximum SD values of *GT*_1_, *GT*_2_, and *GT*_3_. *GT*_*th*_ shows 63.1% of pre-symptomatic contagions.

### ***R***_***0***_*** from r***

In theory, the basic reproduction number *R*_0_ can be estimated as far as the intrinsic growth rate *r*, and the distributions of both the latent and infectious periods are known^26,32–34^. The latent period accounts for the period during which an infected individual cannot infect other individuals. It is observed in diseases for which the infectious period starts around the end of the incubation period, as happened with influenza^35^ and SARS^36^. However, from Fig. 3 it is inferred that COVID-19 is transmissible from the moment of infection, and we will assume a null latent period. Then, if the GT follows a gamma distribution, *R*_*0*_ can be estimated from the formulation of Anderson and Watson^32^, which was adapted to null latent periods by Yan^26^ as

4 $$ R_{0} = \frac{{mean_{GT} }}{{1 - \left( {1 + mean_{GT} \cdot r \cdot \frac{1}{{shape_{GT} }}} \right)^{{ - shape_{GT} }} }} \cdot r, $$

where *mean*_*GT*_ is the mean GT and *shape*_*GT*_ is one of the two parameters defining the gamma distribution, which can be estimated as

5 $$ shape_{GT} = \frac{{\left( {mean_{GT} } \right)^{2} }}{{\left( {SD_{GT} } \right)^{2} }}. $$

For *GT*_*th*_, we get *R*_0_ = 1.50 (CI = [1.41, 1.61]) for REMEDID *I*(*n*) and *R*_0_ = 1.76 (CI = [1.60, 1.94]) for official *I*(*n*). For the other three GT distributions, *R*_0_ ranges from 1.39 (CI = [1.27, 1.58]) to 1.51 (CI = [1.34, 1.80]) for REMEDID *I*(*n*) and from 1.59 (CI = [1.40, 1.88]) to 1.78 (CI = [1.51, 2.23]) for official *I*(*n*) (Table 1). In all cases, *R*_0_ from *GT*_*th*_ are within those from the three known GT distributions and indistinguishable from them within the error estimates. The lower (upper) bound of the CI is estimated as the minimum (maximum) *R*_0_ obtained from all the possible combinations of 100 evenly spaced values covering the CI of *r*, *mean*_*GT*_ and *SD*_*GT*_. Then, following the Bonferroni correction, the reported CI present at least a 85% of confidence level for *GT*_1_, *GT*_2_, and *GT*_3_, but it cannot be assured for *GT*_*th*_ since the CI of its SD is unknown. In general, all these *R*_0_ estimates are lower than those summarised by Park et al.^20^.

**Table 1:** *R*_0_ and HIT values of the ancestral SARS-CoV-2 variant estimated from *GT*_1_, *GT*_2_, *GT*_3_, and *GT*_*th*_, and REMEDID and official infections. For *date*_0_, “Dec.” means December 2019, and “Jan.” means January 2020.

Generation time	PDF gamma distribution		*R*_0_ (conservative CI) for Eqs. (4) and (6); *R*_0_/*date*_0_ (conservative CI) for Dyn. model			HIT from Eq. (2) in percentage (conservative CI)	
	Mean	SD		REMEDID [r = 0.1592]	Official data [r = 0.2322]	REMEDID	Official data
*GT*_1_: Ganyani et al. (2020), Singapore	5.20	1.72	Equation (4)	1.51 (1.34, 1.80)	1.78 (1.51, 2.23)	33.9 (25.6, 44.5)	43.7 (33.7, 55.0)
			Equation (6)	2.21 (1.59, 2.95)	3.11 (1.84, 4.90)	54.7 (37.1, 66.1)	67.8 (45.7, 79.6)
			Dyn. model	2.85/13 Dec (2.05/16 Dec, 3.25/13 Dec)	2.41/1 Jan (1.80/1 Jan, 2.91/1 Jan)	64.9 (51.2, 69,2)	58.5 (44.4, 65.5)
*GT*_2_: Ganyani et al., (2020), Tianjin	3.95	1.51	Equation (4)	1.39 (1.27, 1.58)	1.59 (1.40, 1.88)	28.1 (21.3, 36.7)	37.0 (28.4, 46.7)
			Equation (6)	1.82 (1.48, 2.19)	2.36 (1.69, 3.16)	45.2 (32.4, 54.3)	57.6 (40.7, 68.4)
			Dyn. Model	2.34/14 Dec (1.90/16 Dec, 2.76/12 Dec)	2.01/1 Jan (1.68/1 Jan, 2.33/31 Dec)	57.3 (47.4, 63.8)	50.2 (40.5, 57.1)
*GT*_3_: Ng et al., (2021), Singapore	3.44	2.39	Equation (4)	1.42 (1.28, 1.58)	1.62 (1.41, 1.86)	29.7 (21.9, 36.7)	38.4 (29.0, 46.3)
			Equation (6)	1.63 (1.43, 1.90)	1.97 (1.59, 2.54)	38.5 (30.0, 47.3)	49.1 (37.1, 60.7)
			Dyn. Model	2.08/15 Dec (1.86/17 Dec, 2.42/14 Dec)	1.81/1 Jan (1.64/1 Jan, 2.07/1 Jan)	51.9 (46.2, 58.7)	44.8 (39.0, 51.7%)
*GT*_*th*_: theoretical	4.99	1.88	Equation (4)	1.50 (1.41, 1.61)	1.76 (1.60, 1.94)	33.3 (28.8, 38.0)	43.2 (37.6, 48.6)
			Equation (6)	2.12 (1.81, 2.48)	2.92 (2.28, 3.75)	52.9 (44.8, 59.7)	65.8 (56.1, 73.4)
			Dyn. model	2.71/13 Dec (2.33/14 Dec, 3.15/12 Dec)	2.32/1 Jan (2.01/1 Jan, 2.67/1 Jan)	63.1 (57.1, 68.3)	56.9 (50.2, 62.5)

Lower (higher) bound of any *R*_0_ confidence interval (CI) is estimated conservatively as the minimum (maximum) of the *R*_0_ estimated from all the combinations of 100 evenly spaced values covering the CI of each of the involved parameters. *R*_0_ estimates for alpha and delta variants are obtained increasing these *R*_0_ values on 70% and 189%, respectively. The associated HIT values are obtained from the new *R*_0_ values through Eq. (1).

Alternatively, *R*_0_ can be estimated by applying the Euler–Lotka equation^29,33^,

6 $$ R_{0} = \frac{1}{{\mathop \smallint \nolimits_{0}^{ + \infty } e^{ - rt} \cdot f_{GT} \left( t \right)dt}}. $$

In this case, we get values closer to previous estimates^20^. In particular, for *GT*_*th*_, we get *R*_0_ = 2.12 (CI = [1.81, 2.48]) for REMEDID *I*(*n*) and *R*_0_ = 2.92 (CI = [2.28, 3.75]) for official *I*(*n*). For the other three GT distributions, *R*_0_ ranges from 1.63 (CI = [1.43, 1.90]) to 2.21 (CI = [1.59, 2.95]) for REMEDID *I*(*n*) and from 1.97 (CI = [1.59, 2.54]) to 3.11 (CI = [1.84, 4.90]) for official *I*(*n*) (Table 1). The CI are estimated as in Eq. (4).

### R_0_ from a dynamical model

We designed a dynamic model with Susceptible-Infected-Recovered (SIR) as stocks that accounts for the infectiousness of the infectors. Such a model is a generalisation of the Susceptible-Exposed-Infected-Recovered (SEIR) model^37^. Births, deaths, immigration and emigration are ignored, which seems reasonable since the timescale of the outbreak is too short to produce significant demographic changes. For the sake of simplicity, the recovered stock includes recoveries and fatalities, and it is denoted as *R*(*t*). A random mixing population is assumed, that is a population where contacts between any two people are equally probable. Time is discretized in days, so the real time variable *t* is replaced by the integer variable *n*. As a consequence, the derivatives in the differential equations defining the dynamic model explained below are discrete derivatives.

The size of the population is fixed at *N* = 100,000, and then, for any day *n* we get

7 $$ \tilde{S}\left( n \right) + \left( {\mathop \sum \limits_{k = 0}^{20} \tilde{I}\left( n-k \right)} \right) + \tilde{R}\left( n \right) = N, $$

where $\tilde{S}\left( n \right)$, $\tilde{I}\left( n \right)$, and $\tilde{R}\left( n \right)$ are the discretized versions of *S*(*t*), *I*(*t*), and *R*(*t*) and $\tilde{I}$ is assumed to be null for negative integers. The summation is a consequence of the infectiousness, which is approximated according to the GT, whose PDF is discretized as

8 $$ \widetilde{{f_{GT} }}\left( n \right) = \mathop \smallint \limits_{n - 1}^{n} f_{GT} \left( t \right) dt, $$

from *n* = 1 to 20. Figure 3 shows $\widetilde{{f_{GT} }}\left( n \right)$ for *GT*_*th*_. Truncating at *n* = 20 accounts for 99.99% of the area below the PDF of all the GT. Then, an infected individual at day *n*_0_ is expected to produce on average

9 $$ \widetilde{{R_{e} }}\left( {n_{0} + n} \right) \cdot \widetilde{{f_{GT} }}\left( n \right) $$

infections *n* days later, where $\widetilde{{R_{e} }}\left( n \right)$ is the discretized version of *R*_*e*_(*t*). From this expression, it is obvious that values of $\widetilde{{R_{e} }}\left( n \right) < 1$ will produce a decline of infections. Conversely, infections at day *n*_0_ are produced by all individuals infected during the previous 20 days as

10 $$ \tilde{I}(n_{0} ) = \tilde{R}_{e} \left( {n_{0} } \right) \cdot \left( {\mathop \sum \limits_{n = 1}^{20} \tilde{I}\left( {n_{0} - n} \right) \cdot \widetilde{{f_{GT} }}\left( n \right)} \right), $$

whose continuous version has been reported in previous studies^29,38^. The expression in brackets is called total infectiousness of infected individuals at day *n*_0_^39^. According to Eq. (1), Eq. (10) can be expressed in terms of *R*_0_ as

11 $$ \tilde{I}(n_{0} ) = R_{0} \cdot \frac{{\tilde{S}\left( {n_{0} } \right)}}{N} \cdot \left( {\mathop \sum \limits_{n = 1}^{20} \tilde{I}\left( {n_{0} - n} \right) \cdot \widetilde{{f_{GT} }}\left( n \right)} \right). $$

As we want a dynamic model capable of providing $\tilde{I}\left( {n_{0} } \right)$ from the stocks at time step *n*_0_ − 1, we replaced $\tilde{S}\left( {n_{0} } \right)$ by $\tilde{S}\left( {n_{0} - 1} \right)$ in Eq. (11). This assumption makes sense in a discrete domain since the infections at time *n*_0_ take place in the susceptible population at time *n*_0_ − 1. Then, assuming that all stocks are set to zero for negative integers, our dynamic model can be expressed in terms of Eq. (7) and the following differential equations:

12 $$ \delta \tilde{I}(n_{0} ) = R_{0} \cdot \frac{{\tilde{S}\left( {n_{0} - 1} \right)}}{N} \cdot \left( {\mathop \sum \limits_{n = 1}^{20} \tilde{I}\left( {n_{0} - n} \right) \cdot \widetilde{{{\text{f}}_{GT} }}\left( n \right)} \right) - \tilde{I}(n_{0} - 1), $$

13 $$ \delta \tilde{S}\left( {n_{0} } \right) = {-}\tilde{I}\left( {n_{0} } \right), $$

14 $$ \delta \tilde{R}\left( {n_{0} } \right) = \tilde{I}\left( {n_{0} - 21} \right), $$

where $\delta \tilde{I}$, $\delta \tilde{S}$, and $\delta \tilde{R}$ are the (discrete) derivatives of $\tilde{I}$, $\tilde{S}$, and $\tilde{R}$, respectively. Applying the initial conditions $\tilde{S}\left( 0 \right) = N - 1$, $\tilde{I}\left( 0 \right) = 1$, and $\tilde{R}\left( 0 \right) = 0$, it is assumed that the outbreak was produced by only one infector. The latter is not true in Spain, since several independent introductions of SARS-CoV-2 were detected^40^. However, for modelling purposes it is equivalent to introducing a single infection at day 0 or *M* infections produced by the single infection *n* days later. Then, the date of the initial time *n* = 0 is accounted as a parameter *date*_0_, which is optimised, as well as *R*_0_, to minimise the root-mean square of the residual between the model simulated $\tilde{I}\left( n \right)$ and the REMEDID and official *I*(*n*) for the period from *date*_0_ to *n*_0_.

**Figure 4:**
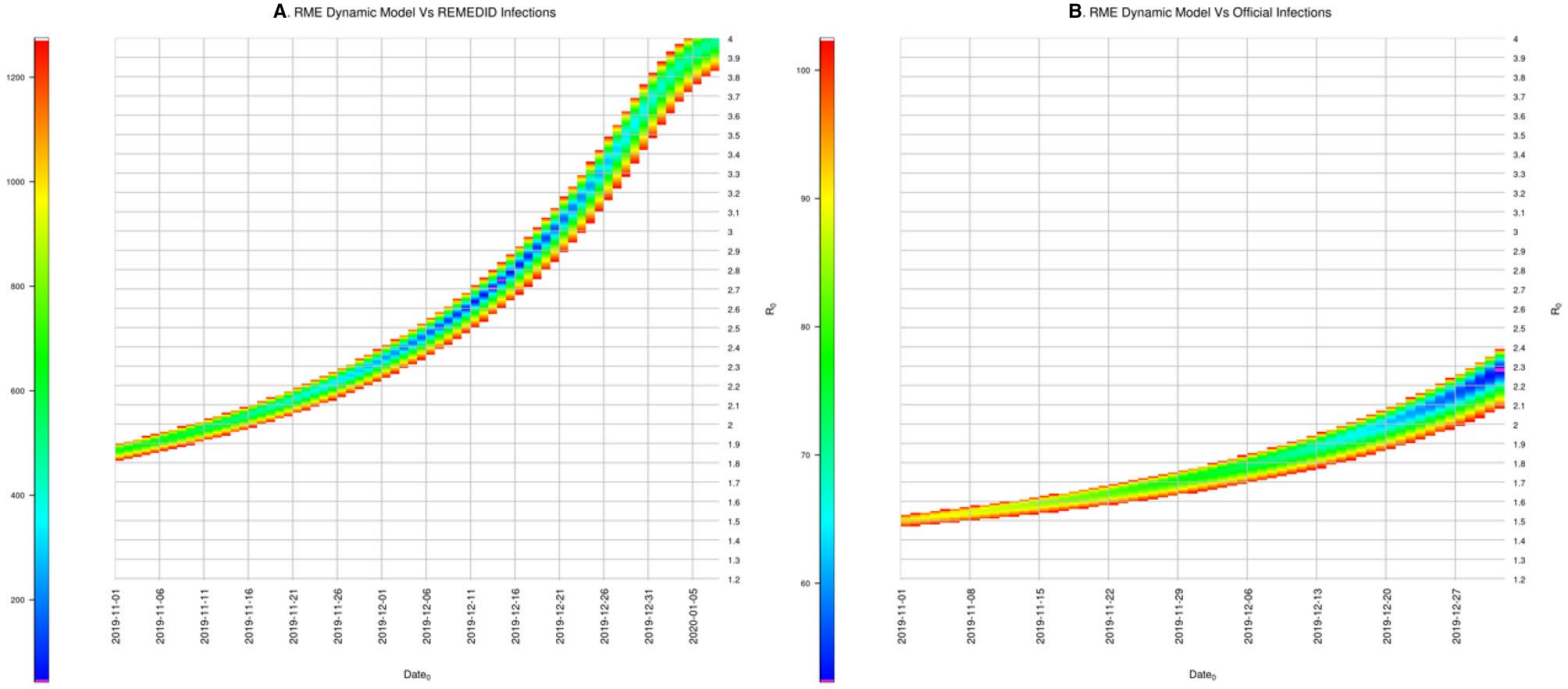
Root-mean square (RMS) of the residuals between infections from the model, which depends on *date*_0_ (x-axis) and *R*_0_ (y-axis), and REMEDID (from MoMo ED) and official infections. Parameters optimizing the model are highlighted in purple. RMS larger than 1275 (left panel) and 103 (right panel) are saturated in white.

The model was implemented in Stella Architect software v2.1.1 (www.iseesystems.com) and exported to R software v4.1.1 with the help of *deSolve* (v1.28) and *stats* (v4.1.1) packages, and the Brent optimisation algorithm was implemented. For REMEDID *I*(*n*) and *GT*_*th*_, we obtained *date*_0_ = 13 December 2019 and *R*_0_ = 2.71 (CI = [2.33, 3.15]). Optimal solutions combine lower/higher *R*_0_ and earlier/later *date*_0_ (Fig. 4), which highlights the importance of providing an accurate first infection date to estimate *R*_0_. When the other three GT distributions were considered, we obtained similar *date*_0_, ranging from 12 to 17 December 2019, and *R*_0_ values ranging from 2.08 (CI = [1.86, 2.42]) to 2.85 (CI = [2.05, 3.25]; see Table 1). For official infections, *date*_0_ was set to 1 January 2020 for all cases, and *R*_0_ ranged from 1.81 (CI = [1.64, 2.07]) to 2.41 (CI = [1.80, 2.91]). The CI are estimated as in Eq. (4).

## Herd immunity threshold and discussion

HIT of the ancestral variant was estimated from *R*_0_ via Eq. (2) and values are shown in Table 1, which range between 28.1 (CI = [21.3, 36.7]) and 64.9% (CI = [51.2, 69.2]) for REMEDID *I*(*n*) (hereafter HIT_R_), and between 37.0 (CI = [28.4, 46.7]) and 67.8% (CI = [45.7, 79.6]) for official *I*(*n*) (Hereafter HIT_O_). The differences between the estimations are determined by three key factors: (1) source/quality of data; (2) GT distribution; and (3) methodology to estimate *R*_0_.

In general, official infection data are of poor quality, but if death records and seroprevalence studies were available, the REMEDID algorithm would provide more reliable infections time series^23^. The maximum difference between HIT_R_ and HIT_O_ is 13.1 percentage points, corresponding to the Eq. (6) estimate, although such difference is not significant within the errors estimates. Moreover, official data vary depending on the date of publication. For example, the maximum HIT_O_ is 67.7%. from data available in February 2021, and 80.1% from data available a year before, in March 2020. The latter is similar to the 80.7% published by Kwok et al.^41^ in March 2020, which was obviously based on data available at that time. The February 2021 version of the data is more realistic than the March 2020 one, and the REMEDID-derived infections are more realistic than both of them^23^. In consequence, results based on REMEDID data should be more reliable.

The most influential factor for estimating the HIT is the methodology to estimate *R*_0_, which may produce differences of ~ 30 percentage points for HIT_R_ and ~ 20 points for HIT_O_ for the same dataset and GT distribution. Such differences are significant within the error estimates for all GT in HIT_R_ and only for *GT*_*th*_ in HIT_O_. For each GT, the lowest HIT values were obtained from Eq. (4), but the largest HIT_R_ and HIT_O_ are obtained from the dynamic model and Eq. (6), respectively. The CI from Eq. (6) and the dynamic model are longer than those from Eq. (4), meaning that the former are more sensitive to errors in the involved parameters. Moreover, the largest errors are obtained from Eq. (6) for both HIT_R_ and HIT_O_, although they are larger for HIT_O_. It means that Eq. (6) is the methodology most sensitive to parameters and data quality. In general, results from Eq. (6) are reconcilable with the other two within the error estimates, but Eq. (4) and the dynamic model are only reconcilable for official data (Table 1).

The selection of a GT produces HIT differences up to 6 percentage points when *R*_0_ is estimated from Eq. (4); 18.7 from Eq. (6); and 13.7 from the dynamic model, although in no case are significantly different within the error estimates. It is more difficult to estimate the GT than the serial interval. For that reason, many studies approximate the GT by a serial interval (e.g.^39,41^). However, though GT and serial interval have the same mean, serial interval presents a larger variance^30^, which will underestimate *R*_0_ when using Eq. (6) ^29^. HIT values from Eq. (4) for any GT are included in the CI obtained for the other GT. On the contrary, although all the CI estimated from Eq. (6) overlap among them, only some HIT values are included in the CI estimated for other GT. This is also the situation for the HIT estimated from the dynamic model.

The influential factors should be kept in mind when interpreting *R*_0_ estimates. For example, Locatelli et al.^21^ estimated an average *R*_0_ of 2.2 (CI = [1.9, 2.6]) for Western Europe by using official data available in September 2020, a theoretical approximation of GT, and Eq. (6). For any GT in Table 1 it can be observed that: (1) official data produces the highest *R*_0_ values for Eq. (6) with respect to Eq. (4), and the dynamic model; and that (2) the more realistic REMEDID data also produces lower *R*_0_ values when Eq. (6) is used. Then, it could be conjectured that the *R*_0_ reported by Locatelli et al.^21^ is in the upper bound of all the possible *R*_0_ estimates for Western Europe.

In summary, accurately estimating HIT is quite complicated. In any case, assuming that REMEDID-derived infection data are more accurate than official data, 70% seems to be a good upper bound of HIT for the ancestral variant. However, the upper bound increases to 80% (accounting for the CI) if we rely on official data. Besides, the most important impediment to determine the value of the HIT is that it is variable in time. The more transmissible new SARS-CoV-2 variants present higher *R*_*e*_, and in consequence a higher (theoretical) associated *R*_0_ and higher HIT values. For example, the B.1.1.7 lineage (also known as alpha variant), which was first detected in England in September 2020^42^, and thereafter rapidly spread around the world. In Spain, at the beginning of January 2021, the alpha variant was ~ 30% of the circulating SARS-CoV-2 variants, but it was over 80% from March to May 2021^43^. On the other hand, the B.1.617.2 lineage (also known as delta variant), first detected in India in December 2020^44^, has represented over 95% of the SARS-CoV-2 variants in Spain from late July to at least up to October 2021^43^. Both alpha and delta displaced the previous variants because of their higher transmissibly. Although the *R*_0_ cannot be directly estimated for these variants since they appeared in the middle of the pandemic, the *R*_*e*_ can. It has been estimated that the *R*_*e*_ of the alpha variant is ~ 70% higher than in previous existing variants^45^. On the other hand, the *R*_*e*_ of the delta variant is also ~ 70% higher than in alpha variant^46^. Following Eq. (1) and assuming that the variations of *R*_*e*_ from one variant to another are not produced by changes in the control measures, it can be inferred that the *R*_0_ of the alpha and delta variants are 70% and 189% higher than the ancestral variant, respectively. Therefore, if we take the highest estimate of *R*_0_ in Table 1 (*R*_0_ = 3.11, CI = [1.84, 4.90]; for *GT*_1_, Eq. (6), official data) as an upper bound of the *R*_0_ of the ancestral variant, we get that 8.99 (CI = [5.32, 14.16]) is an upper bound estimate of the delta variant *R*_0_. In that case, we can conclude that an upper bound of the HIT at present in Spain is 88.9% (CI = [81.2, 92.9]). For a more realistic upper bound, we could alternatively take the maximum *R*_0_ for REMEDID data in Table 1 (*R*_0_ = 2.85, CI = [2.05, 3.25]; for *GT*_1_, dynamic model) as an upper bound, which would produce *R*_0_ = 8.24 (CI = [5.92, 9.39]) and HIT = 87.9% (CI = [83.1, 89.4%]) as upper bound for the delta variant, in agreement with previous estimates^46^. Then, a HIT of 90% seems to be realistic for Spain with a predominant delta variant as in October 2021.

The presented results are valid for a randomly mixing population with a spread dynamic similar to Spain as a whole. However, even Spanish regions show different dynamics between themselves^23^, which may lead to specific HIT values for each region. It should be kept in mind that none of the three vaccines administered in Spain are able to completely prevent the transmission of the virus. Then, even with a 90% of the population vaccinated, the HIT will probably not be reached. However, it is true that the risk of infection is significatively reduced for vaccinated susceptible individuals^6,7^, which directly reduces the *R*_0_. Besides, in case of infection, the transmission of the virus is also reduced^8–10^, which modifies the associated GT, and reduces the *R*_0_ and the HIT of a vaccinated population. So, even if transmission is not completely prevented by vaccines, the greater the proportion of the vaccinated population, the lower the HIT. Therefore, it is expected that the HIT of a highly vaccinated population will be below the estimated 90% upper bound. However, all this may change with the emergence and spread of new variants with re-infection capacity^47^. In any case, even if the HIT is reached, it will not be the panacea. First, if HIT is reached in most places in a country but there are some specific regions or population subgroups in a region with a percentage of immune individuals below HIT, local outbreaks will be possible for those regions or subgroups. Second, the final size of an epidemic in a randomly mixing population with HIT = 70% and 90% is reached at 95.9% and 99.9% of infections, respectively^15,37^. This means that if the ancestral variant would have not been replaced, the decreasing rate of infections after reaching a HIT of 70% may still produce a non-negligible 25.9% of infections, that is 12.2 million infections in Spain. Third, interpretation of HIT values must be done carefully and overoptimistic messages should be avoided as has been learnt from Manaus in the Brazilian state of Amazonas. In October 2020, it was thought that Manaus had reached the HIT with 76% of infected population^48^, which led to a relaxation of the control measures. However, either because the percentage of infected population was not accurately estimated or because the new SARS-CoV-2 P.1 variant was capable of re-infecting, Manaus had a second wave in January 2021 with a higher mortality rate than in the first one^49^. Therefore, health authorities should strictly ensure an adaptive and proactive management of the new situation after theoretical herd immunity is reached.

## References

[1] Our world in data, https://ourworldindata.org/coronavirus-data-explorer). Accessed 1 Apr 2021.

[2] Defunciones según la Causa de Muerte—Avance enero-mayo de 2019 y de 2020. Notas de prensa del Instituto Nacional de Estadística (2020). https://www.ine.es/dyngs/INEbase/es/operacion.htm?c=Estadistica_C&cid=1254736176780&menu=ultiDatos&idp=1254735573175. Accessed 16 Feb 2021.

[3] Kung S, Doppen M, Black M, Braithwaite I, Kearns C, Weatherall M, Beasley R, Kearns N (2021). Underestimation of COVID-19 mortality during the pandemic. ERJ Open Res..

[4] Modi C, Böhm V, Ferraro S, Stein G, Seljak U (2021). Estimating COVID-19 mortality in Italy early in the COVID-19 pandemic. Nat. Commun..

[5] European Medicines Agency, https://www.ema.europa.eu/en/human-regulatory/overview/public-health-threats/coronavirus-disease-covid-19/treatments-vaccines/covid-19-vaccines. Accessed 1 Apr 2021.

[6] Hall VJ, Foulkes S, Saei A, Andrews N, Oguti B (2021). COVID-19 vaccine coverage in health-care workers in England and effectiveness of BNT162b2 mRNA vaccine against infection (SIREN): A prospective, multicentre, cohort study. The Lancet..

[7] Thompson MG, Burgess JL, Naleway AL (2021). Interim estimates of vaccine effectiveness of BNT162b2 and mRNA-1273 COVID-19 vaccines in preventing SARS-CoV-2 infection among health care personnel, first responders, and other essential and frontline workers—Eight US Locations, December 2020–March 2021. MMWR Morb. Mortal. Wkly. Rep..

[8] Levine-Tiefenbrun M, Yelin I, Katz R, Herzel E, Golan Z (2021). Initial report of decreased SARS-CoV-2 viral load after inoculation with the BNT162b2 vaccine. Nat. Med..

[9] Emary KRW, Golubchik T, Aley PK, Ariani CV, Angus B (2021). Efficacy of ChAdOx1 nCoV-19 (AZD1222) vaccine against SARS-CoV-2 variant of concern 202012/01 (B.1.1.7): An exploratory analysis of a randomised controlled trial. The Lancet..

[10] Shah ASV, Gribben C, Bishop J, Hanlon P, Caldwell D (2021). Effect of vaccination on transmission of SARS-CoV-2. N. Engl. J. Med..

[11] Fine P, Eames K, Heymann DL (2011). “Herd immunity”: A rough guide. Clin. Infect. Dis..

[12] Anderson RM, May RM (1991). Infectious Diseases of Humans: Dynamics and Control.

[13] Hannon B, Ruth M (2009). Dynamic Modeling of Diseases and Pests.

[14] Heesterbeek JAP (2002). A brief history of R_0_ and a recipe for its calculation. Acta. Biotheor..

[15] Kermack WO, McKendrick AG (1927). A contribution to the mathematical theory of epidemics. Proc. R. Soc. Lond. A..

[16] Keeling M, Rohani P (2008). Modeling Infectious Diseases in Humans and Animals.

[17] Coburn BJ, Wagner BG, Blower S (2009). Modeling influenza epidemics and pandemics: Insights into the future of swine flu (H1N1). BMC Med..

[18] Bauch CT, Lloyd-Smith JO, Coffee MP, Galvani AP (2005). Dynamically modeling SARS and other newly emerging respiratory illnesses: Past, present, and future. Epidemiology.

[19] Breban R, Riou J, Fontanet A (2013). Interhuman transmissibility of Middle East respiratory syndrome coronavirus: Estimation of pandemic risk. Lancet.

[20] Park M, Cook AR, Lim JT, Sun Y, Dickens BL (2020). A systematic review of COVID-19 epidemiology based on current evidence. J. Clin. Med..

[21] Locatelli I, Trächsel B, Rousson V (2021). Estimating the basic reproduction number for COVID-19 in Western Europe. PLoS ONE.

[22] Instituto de Salud Carlos III, https://cnecovid.isciii.es/covid19/#documentaci%C3%B3n-y-datos. Accessed 12 Feb 2021.

[23] García-García D, Vigo MI, Fonfría ES, Herrador Z, Navarro M, Bordehore C (2021). Retrospective methodology to estimate daily infections from deaths (REMEDID) in COVID-19: The Spain case study. Sci. Rep..

[24] European Mortality Monitoring surveillance system, http://www.euromomo.eu. Accessed 16 Feb 2021.

[25] Pollán M, Pérez-Gómez B, Pastor-Barriuso R, Oteo J, Hernán MA, Pérez-Olmeda M (2020). Prevalence of SARS-CoV-2 in Spain (ENE-COVID): A nationwide, population-based seroepidemiological study. The Lancet..

[26] Yan P (2008). Separate roles of the latent and infectious periods in shaping the relation between the basic reproduction number and the intrinsic growth rate of infectious disease outbreaks. J. Theor. Biol..

[27] Ganyani T, Kremer C, Chen D, Torneri A, Faes C, Wallinga J (2020). Estimating the generation interval for coronavirus disease (COVID-19) based on symptom onset data. Euro Surveill..

[28] Ng S, Kaur P, Kremer C, Tan W, Tan A, Hens N (2021). Estimating transmission parameters for COVID-19 clusters by using symptom onset data, Singapore, January–April 2020. Emerg. Infect. Dis..

[29] Britton T, Tomba GS (2019). Estimation in emerging epidemics: Biases and remedies. J. R. Soc. Interface.

[30] Lehtinen S, Ashcroft P, Bonhoeffer S (2021). On the relationship between serial interval, infectiousness profile and generation time. J. R. Soc. Interface..

[31] Fonfría ES, Vigo MI, García-García D, Herrador Z, Navarro M, Bordehore C (2021). COVID-19 epidemiological parameters for clinical and mathematical modeling: Mini-review and meta-analysis from Asian studies during early phase of pandemic. Front. Med..

[32] Anderson D, Watson R (1980). On the spread of a disease with gamma distributed latent and infectious periods. Biometrika.

[33] Wallinga J, Lipsitch M (2007). How generation intervals shape the relationship between growth rates and reproductive number. Proc. R. Soc. B..

[34] Roberts MG, Heesterbeek JAP (2007). Model-consistent estimation of the basic reproduction number from the incidence of an emerging infection. J. Math. Biol..

[35] Lau LL, Cowling BJ, Fang VJ, Chan KH, Lau EHY, Lipsitch M (2010). Viral shedding and clinical illness in naturally acquired influenza virus infections. J Infect Dis..

[36] Peiris JS, Chu CM, Cheng VC, Chan KS, Hung IFN, Poon LLM (2003). Clinical progression and viral load in a community outbreak of coronavirus-associated SARS pneumonia: A prospective study. Lancet.

[37] Ma J, Earn DJD (2006). Generality of the final size formula for an epidemic of a newly invading infectious disease. Bull. Math. Biol..

[38] Park SW, Sun K, Champredon D, Li M, Bolker BM, Earn DJD, Weitz JS (2021). Forward-looking serial intervals correctly link epidemic growth to reproduction numbers. PNAS.

[39] Cori A, Ferguson NM, Fraser C, Cauchemez S (2013). A new framework and software to estimate time-varying reproduction numbers during epidemics. Am. J. Epidemiol..

[40] Gómez-Carballa A, Bello X, Pardo-Seco J, Pérez del Molino ML, Martinón-Torres F, Salas A (2020). Phylogeography of SARS-CoV-2 pandemic in Spain: A story of multiple introductions, micro-geographic stratification, founder effects, and super-spreaders. Zool. Res..

[41] Kwok KO, Lai F, Wei WI, Wong SYS, Tang JWT (2020). Herd immunity—Estimating the level required to halt the COVID-19 epidemics in affected countries. J. Infect..

[42] Public Health England, “Investigation of novel SARS-COV-2 variant: Variant of Concern 202012/01” (2020); www.gov.uk/government/publications/investigation-of-novel-sars-cov-2-variant-variant-of-concern-20201201.

[43] Actualización de la situación epidemiológica de las variantes de SARS-CoV-2 de preocupación (VOC) e interés (VOI) en salud pública en España, 18 de octubre de 2021, https://www.mscbs.gob.es/profesionales/saludPublica/ccayes/alertasActual/nCov/documentos/COVID19_Actualizacion_variantes_20211018.pdf. Accessed 22 Oct 2021.

[44] Cherian S, Potdar V, Jadhav S, Yadav P, Gupta N et al. Convergent evolution of SARS-CoV-2 spike mutations, L452R, E484Q and P681R, in the second wave of COVID-19 in Maharashtra, India. BioRxiv. 2021. Preprint at 10.1101/2021.04.22.440932. 10.3390/microorganisms9071542 PMC8307577 34361977

[45] Davies NG, Abbott S, Barnard RC, Jarvis CI, Kucharski AJ (2021). Estimated transmissibility and impact of SARS-CoV-2 lineage B.1.1.7 in England. Science.

[46] Liu Y, Rocklöv J (2021). The reproductive number of the Delta variant of SARS-CoV-2 is far higher compared to the ancestral SARS-CoV-2 virus. J. Travel Med..

[47] Uriu K, Kimura I, Shirakawa K, Takaori-Kondo A, Nakada T, Kaneda A, The genotype to phenotype Japan (G2P-Japan) consortium, So Nakagawa, Kei Sato. Ineffective neutralization of the SARS-CoV-2 Mu variant by convalescent and vaccine sera. bioRxiv; 2021. 10.1101/2021.09.06.459005 10.1056/NEJMc2114706 PMC8609602 34731554

[48] Buss LF, Prete CA, Abrahim CMM, Mendrone A, Salomon T, de Almeida-Neto C (2021). Three-quarters attack rate of SARS-CoV-2 in the Brazilian Amazon during a largely unmitigated epidemic. Science.

[49] Taylor L (2021). Covid-19: Is Manaus the final nail in the coffin for natural herd immunity?. BMJ.

